# Paeoniflorin Rescued MK-801-Induced Schizophrenia–Like Behaviors in Mice via Oxidative Stress Pathway

**DOI:** 10.3389/fnut.2022.870032

**Published:** 2022-04-27

**Authors:** Jia-Quan Liang, Xi Chen, Yong Cheng

**Affiliations:** ^1^The Third People's Hospital of Foshan, Foshan, China; ^2^Center on Translational Neuroscience, School of Pharmacy, Minzu University of China, Beijing, China

**Keywords:** schizophrenia, paeoniflorin, MK-801, olanzapine, oxidative stress

## Abstract

Schizophrenia (SCZ) affects approximately 1% population worldwide, and the first-line antipsychotics have partial reactivity or non-reactivity with side effects. Therefore, there is an urgent need to find more effective drugs. Paeoniflorin (PF) is the main effective component of traditional Chinese medicine from white peony, red peony and peony bark, which acts as a neuroprotective agent. The purpose of this study was to investigate whether PF can rescue MK-801 induced schizophrenia-like behavior in mice. Our results demonstrated that intragastric administration of PF ameliorated MK-801 induced schizophrenia–like behaviors in mice as demonstrated by prepulse inhibition of acoustic startle response, fear conditioning test for memory and open field test for activity. In contrast, the first-line antipsychotics-olanzapine reversed the prepulse inhibition deficits and hyperactivities, but not memory deficits, in the model mice. Further analysis showed that PF reduced oxidative stress in the MK-801-treated mice, as evidenced by the increased superoxide dismutase levels and decreased malondialdehyde levels in the blood of the model mice. In addition, PF treatment inhibited the expression of the apoptotic protein Bax and restored the expression of tyrosine hydroxylase in the brains of the model mice. *in vitro* data indicated that PF protected against oxidative stress induced neurotoxicity in the primary cultured hippocampal neurons. In conclusion, our results were the first to provide evidence that PF rescued schizophrenia-like behaviors (both positive symptoms and cognitive impairments) in rodents through oxidative stress pathway, and therefore provide a novel strategy for treatment of SCZ. However, more pre-clinical and clinical research are needed to translate the present findings into clinics for a treatment of schizophrenia.

## Introduction

Schizophrenia (SCZ) is a neurodevelopmental disorder underpinned by complex interactions between genetic and environmental factors. Its annual prevalence was as high as 0.6% (1), which has a far-reaching impact on personal life and society, and it is easy to lead to lifelong disability. At present, the treatment of SCZ is still mainly drug treatment, its curative effect is limited, and adverse drug reactions are difficult to avoid, although it does not make the antipsychotics not being tolerable in most cases (2). Additionally, it has been suggested that identifying different discontinuation patterns for antipsychotics may contribute to optimize treatment selection in SCZ (3). Nevertheless, finding drugs with less adverse reactions and significant curative effect has always been the direction of researchers' unremitting efforts.

Herbal therapy for various diseases has been used for many years in Asia, with low economic cost and few side effects (4). Additionally, both clinical and pre-clinical studies have suggested the beneficial effects of herbal therapy and/or natural products in neuropsychiatric diseases (4, 5). Paeoniflorin (PF) is the main effective component of traditional Chinese medicine of white peony, which is a kind of monoterpenoids (7). Previous studies have shown the neuroprotective effects of PF both *in vitro* and *in vivo*, and the effects were related to its anti-inflammatory and anti-apoptotic properties (7–9). Noticeably, independent groups found that PF exerted anti-depressant-like effects in various animal models of depression over the last several years, these included chronic stress-induced depression in mice and rats (10, 11), menopause depression in ovariectomized rats (12) and post-stroke depression in rats (13). These recent development suggested that PF is a promising therapeutic target for treatment of depression, and may expand it to other psychiatric diseases. However, the potential role of PF in treatment of other major psychiatric diseases is not well explored.

Here we used MK-801 animal model of SCZ to assess the potential therapeutic role of PF in SCZ, and compared it with the first-line antipsychotics-olanzapine. Additionally, given previous studies had shown that there were abnormalities in the oxidation-reduction system in SCZ, and these abnormalities were expected to become new targets for the treatment of SCZ (14, 15). Additionally, Buosi et al. analyzed oxidative stress markers catalase, superoxide dismutase (SOD), glutathione peroxidase, total glutathione and malondialdehyde (MDA), and they showed that SOD levels were lower among schizophrenia patients, while MDA and catalase levels were higher (16). We therefore analyzed several oxidative stress markers after PF treatment in the animal model. We further utilized primary cultured hippocampal neurons to directly evaluate the effects of PF against oxidative stress.

## Materials and Methods

### Primary Cultured Hippocampal Neurons and Treatment

Primary cultured hippocampal neurons were prepared as described previously with modifications (17). Briefly, the hippocampus was dissected and digested by 2 ml 0.25% trypsin for 30 min at 37°C. The tissue was triturated by a pipette to make a homogenous mixture which was then passed through a cell strainer to remove undissociated tissue. We then centrifuged the cell solution and discarded the supernatant, and resuspended the cell pellet in DMEM containing 1 × antibiotics (Penicillin-Streptomycin) and 5% FBS. The primary cultured neurons were subsequently seeded on poly-l-lysine (Sigma, St. Louis, MO) coated plates. The medium was replaced by Neurobasal medium with 2% B27 (Invitrogen, Carlsbad, CA) after plating over-night.

The primary cultured hippocampal neurons (5–7 days of culture) were incubated with synthetic PF (Shanghai Yuanye Bio-Technology Co., Ltd.) at various concentrations (2 μg/ml and 200 ng/ml) for 3 h. The neurons were then treated with 100 μM H_2_O_2_ for 24 h. The cell viability was then detected by water-soluble tetrazolium (WST-1) assay and the degree of cytotoxicity was detected by lactate dehydrogenase (LDH) release assay. The conditional medium from the primary cultured hippocampal neurons after various treatment were collected for oxidative stress marker analysis.

### Animals and Experimental Design

Male C57BL/6 mice (6-week old) were purchased from Vital River (Beijing, China). The animals were raised in a temperature- and humidity-controlled room with a light: dark cycle of 12 h and provided ad libitum access to a standard diet and drinking water. All animal experiments in this study were conducted in accordance with the National Institutes of Health Laboratory Animal Care and Use Guidelines (NIH Publication No. 80-23) and were approved by the Animal Care and Use Committee of Minzu University of China.

**Figure 1:**
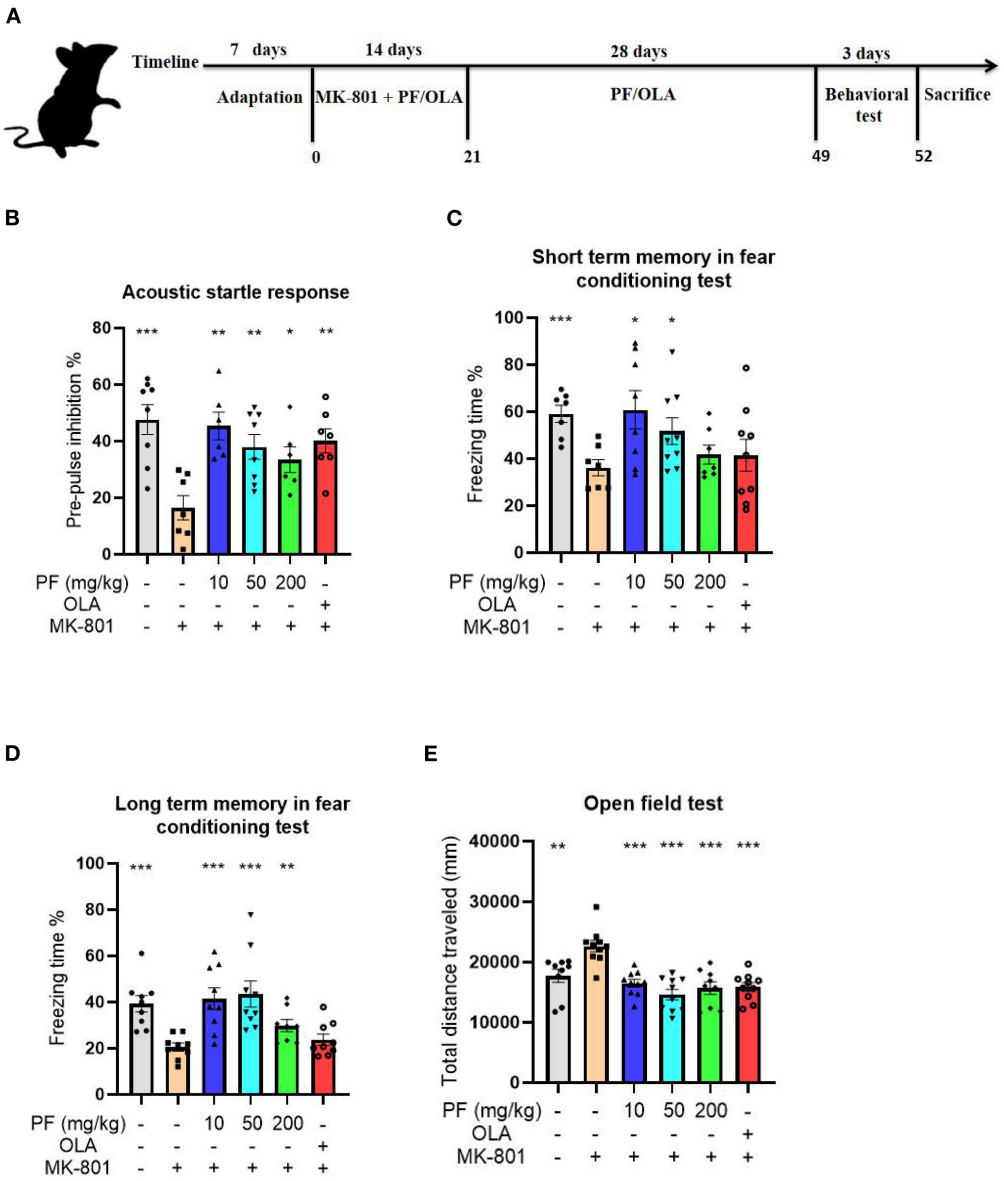
Paeoniflorin (PF) rescued MK-801 induced schizophrenia–like behaviors in mice. **(A)** Schematic representation of experimental design for the MK-801-induced schizophrenia model. **(B)** The PPI deficits observed in the model mice were significantly reversed by PF treatment, and the effects were comparable with the first-line antipsychotics olanzapine on the model mice. **(C)** PF treatment significantly rescued the memory deficits in the MK-801-treated mice. **(D)** The impaired long-term memory in the model mice were significantly improved by treatment of PF. **(E)** The open field test demonstrated that the hyperactivities of the schizophrenia model mice were significantly inhibited by the administration of PF. Additionally, olanzapine also inhibited the hyperactivities of the MK-801-treated mice. Three-way ANOVA statistics: *F*_B_ = 5.749, *P* < 0.001; *F*_C_ = 2.905, *P* = 0.025; *F*_D_ = 6.855, *P* < 0.001; *F*_E_ = 10.87, *P* < 0.001. Values are the mean ± SEM, *n* = 7–10/group. *p < 0.05, **p < 0.01, ***p < 0.001.

After one-week acclimation, the mice were randomly divided into 6 groups with 7–10 mice in each group: control group, SCZ model group, SCZ model plus low-dose PF group (10 mg/kg, orally gavage every day), SCZ model plus medium-dose PF group (50 mg/kg, orally gavage every day), SCZ model plus high-dose PF group (100 mg/kg, orally gavage every day) and SCZ model plus olanzapine group (25 mg/kg). The PF dose and duration of its administration were determined according to a previous literature with modifications (18). The animal model of SCZ was established by intraperitoneal administration of MK-801 for 2 weeks (0.5 mg/kg, every day) in the absence or presence of intragastric administration of PF or olanzapine, and the rescue groups were continued for 4 weeks of PF or olanzapine treatment. The injection schedule of MK-801 was referred to a previous report with modifications (19), and the dose of olanzapine was referred to a previous study with modifications (20). Both PF and olanzapine were prepared in ultrapure water, and MK-801 was prepared in saline. The animal experimental procedure is shown in Figure 1A.

### Behavioral Tests

After drug treatments, all the mice were tested by the prepulse inhibition (PPI) test, open field test and fear conditioning test. The PPI test and open field test were performed according to our previously published papers (6, 21).

For PPI test, briefly, mice were directly placed into the test chamber after treatments. A 60 dB white background noise was presented during the entire testing period. All mice were acclimated to the white noise for 5 min, before receiving five random 120 dB pulses for 20 ms to acquire a stable startle as a baseline. The prepulse inhibition testing phase consisted of three different acoustic stimuli: pulse-only (120 dB for 20 ms), no stimulus (only white noise), prepulse stimuli (75 dB, 10 ms each) followed by a pulse (120 dB for 10 ms). Each stimulus type was presented 10 times with 20-30 s intertrial intervals.

The fear conditioning test was performed using a commercially available system (Tai League Software, Chengdu, China). The protocol followed a previous literature with modifications (22). Briefly, the training session to establish condition fear in mice was 7.2 min long. Mice were first allowed to habituate in the electric-shock chamber for 2 min, and subsequently six 52-s trainings were carried out. Each 52-s training started with a 30-s sound signal, followed by a 2-s footshock (0.2 mA), and then habituated for 20 s. After 24 h (considered as short term memory) and 96 h (considered as long term memory) of trainings, the mice were put into the electric-shock chamber and subjected to the same protocol as the training session, except that without footshock. The percentage of freezing time of mice was recorded to indicate memory function.

### Measurement of Oxidative Stress Markers

Superoxide dismutase (SOD) and malondialdehyde (MDA) activities or levels in the serum of mice or conditional medium from primary cultured neurons were measured by the SOD and MDA kits according to the instructions provided by the manufacturer (Nanjing Jiancheng Bioengineering Institute, Nanjing, China).

### Western Blot Analysis

Western blot was performed as previously described (23). Briefly, protein lysates were prepared by homogenizing the tissues in tissue lysis buffer (Beyotime, China) supplemented with inhibitor Cocktail (Roche, USA) and centrifuged. The total protein concentration was detected by enhanced BCA protein assay kit (Pierce Biotechnology, Rockford, IL). Then 20 μg sample was run on 12% SDS-polyacrylamide gel electrophoresis gels and transferred onto polyvinylidene fluoride membrane (Bio-Rad, USA). After blocking with 5% nonfat milk at room temperature for 1 h, the membrane was incubated with primary antibodies overnight. After washing, the membrane was incubated with fluorescent-conjugated anti-mouse antibody, visualized by the Odyssey infrared imaging system (LI-COR, Lincoln, USA). Monoclonal mouse Bax (1:1000), hydroxylase (TH, 1:1000) and β-Actin (1:10,000) antibodies were from Cell Signaling Technology (Danvers, MA).

### Statistical Analysis

All data analyses were performed using graphpad prism 5.0 software. The results were expressed as Mean ± SEM (standard error of mean). The significance between groups was calculated by three-way analysis of variance (ANOVA) followed by a post hoc test. *P* < 0.05 was considered statistically significant.

## Results

### PF Rescued MK-801 Induced Schizophrenia–Like Behaviors in Mice

The PPI of the acoustic startle response was performed to evaluate the sensorimotor gating disparities of the tested mice. Compared to the saline-treated mice, MK-801-treated mice exhibited significantly decreased PPI of the acoustic startle response (*P* < 0.001). The PPI deficits observed in the model mice were significantly reversed by 10 mg/kg (*P* < 0.01), 50 mg/kg (*P* < 0.01) and 200 mg/kg PF (*P* < 0.05) treatment, and the effects were comparable with the first-line antipsychotics olanzapine on the model mice (*P* < 0.01; Figure 1B).

We next estimated the cognitive impairment in the MK-801-treated mice by the fear conditioning test. Our results showed that the short term memory was significantly impaired in the model mice (*P* < 0.001) when compared with control mice, since MK-801-treated mice exhibited reduced freezing time in the fear conditioning test (Figure 1C). However, 10 mg/kg (*P* < 0.05) and 50 mg/kg (*P* < 0.05), but not 200 mg/kg (*P* >0.05) PF treatment significantly rescued the memory deficits in the MK-801-treated mice (Figure 1C). In contrast, the impaired long-term memory in the model mice were significantly improved by treatment of PF at the dose of 10 mg/kg (*P* < 0.001), 50 mg/kg (*P* < 0.001) and 200 mg/kg (*P* < 0.01; Figure 1D). Noticeably, olanzapine did not improve the cognitive impairments in the MK-801 treated mice, both for short-term memory and long term memory (Figures 1C,D).

We then evaluated the effects of PF treatment on the locomotor activity of the mice. The open field test demonstrated that MK-801-treated mice had significantly increased total distances that the mice moved when compared with control mice (*P* < 0.01), suggesting that the model mice were hyperactive (Figure 1E). However, the hyperactivities of the SCZ model mice were significantly inhibited by the administration of PF at the dose of 10 mg/kg (*P* < 0.001), 50 mg/kg (*P* < 0.001) and 200 mg/kg (*P* < 0.001; Figure 1E). Additionally, olanzapine also inhibited the hyperactivities of the MK-801-treated mice (*P* < 0.001; Figure 1E).

### PF Ameliorated MK-801-Induced Oxidative Stress and Apoptosis *in vivo*

**Figure 2:**
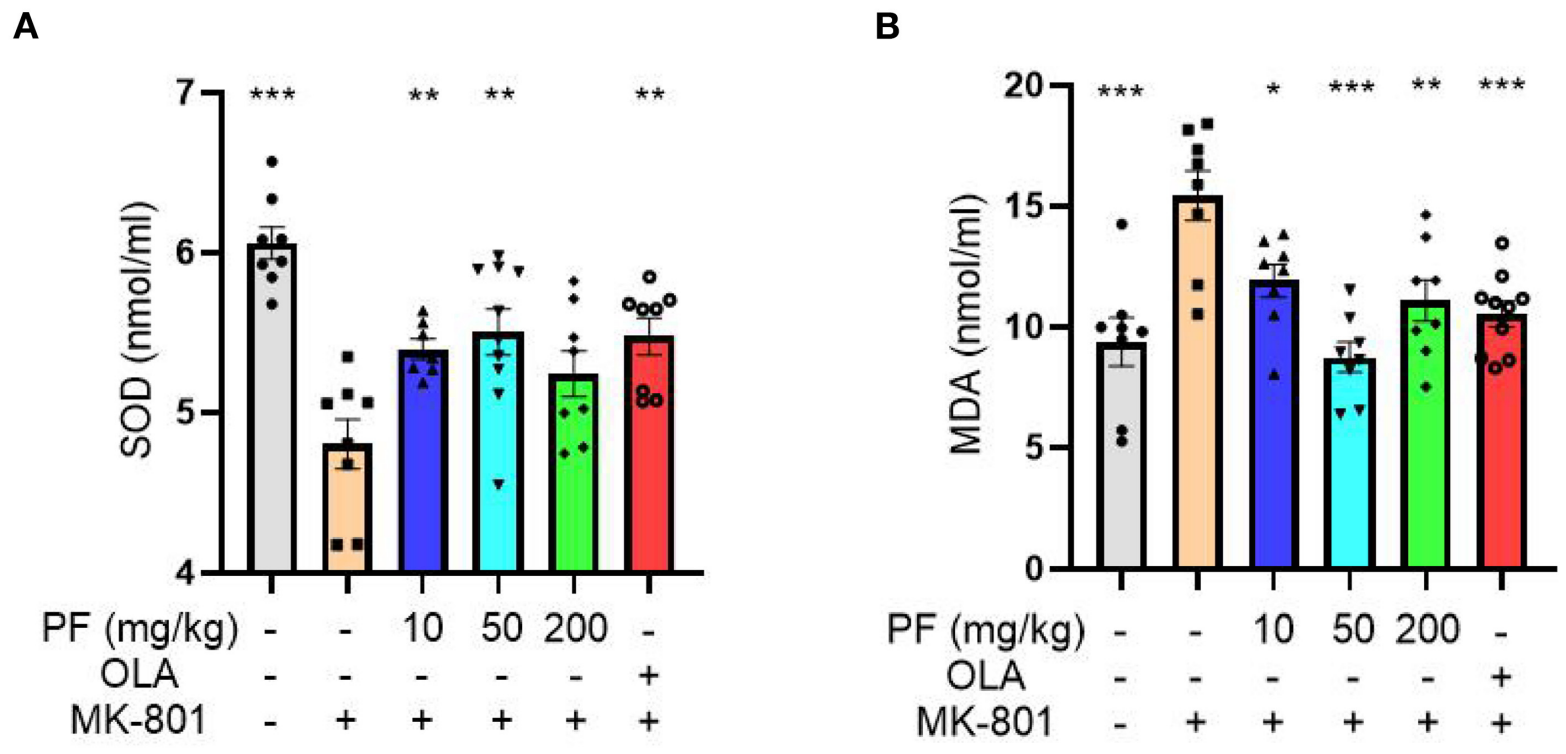
Paeoniflorin (PF) ameliorated MK-801-induced oxidative stress in mice. **(A)** MK-801-treated mice exhibited reduced superoxide dismutase activity in serum (*P* < 0.001), and 10 mg/kg (*P* < 0.01) and 50 mg/kg (*P* < 0.01), but not 200 mg/kg (*P* >0.05) PF administration significantly increased SOD activity in the model mice. **(B)** The enhanced serum MDA content found in the model mice was significantly reduced by the treatment of PF at 10 mg/kg (*P* < 0.05), 50 mg/kg (*P* < 0.001) and 200 mg/kg (*P* < 0.01). Three-way ANOVA statistics: *F*_A_ = 9.849, *P* < 0.001; *F*_B_ = 8.751, *P* < 0.001. Values are the mean ± SEM, *n* = 7–10/group. *p < 0.05, **p < 0.01, ***p < 0.001.

The reduced SOD activity and increased MDA content can be used as biomarkers for oxidative stress-induced damage. MK-801-treated mice exhibited reduced SOD activity in serum (*P* < 0.001), and 10 mg/kg (*P* < 0.01) and 50 mg/kg (*P* < 0.01), but not 200 mg/kg (*P* >0.05) PF administration significantly increased SOD activities in the model mice (Figure 2A). In contrast, the elevated serum MDA content found in the model mice was significantly reduced by the treatment of PF at 10 mg/kg (*P* < 0.05), 50 mg/kg (*P* < 0.001) and 200 mg/kg (*P* < 0.01; Figure 2B). Furthermore, we found that both SOD and MDA aberrations in the MK-801 treated mice were partially restored by olanzapine treatment (Figures 2A,B).

**Figure 3:**
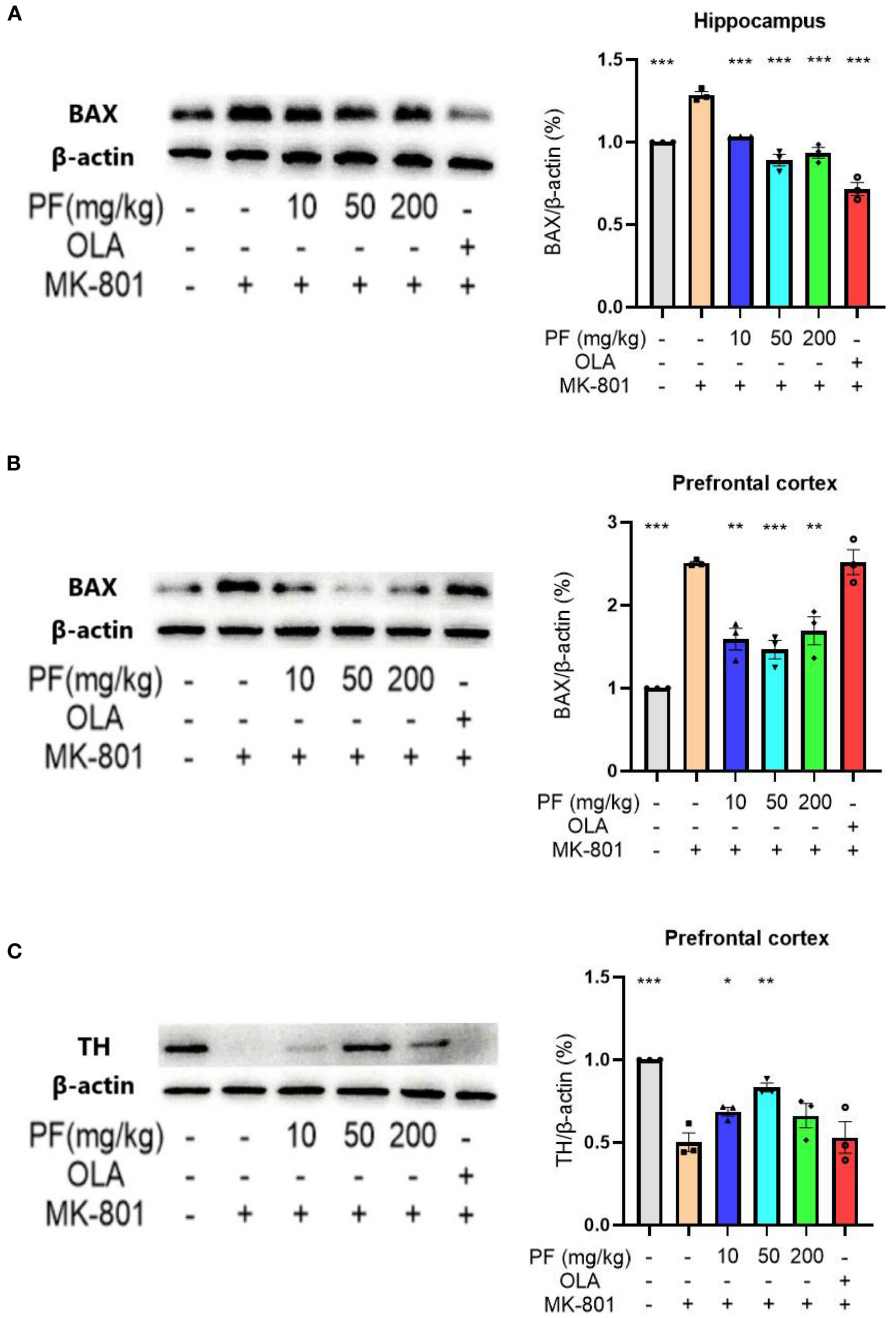
Paeoniflorin (PF) ameliorated MK-801-induced apoptosis in the brains of mice. **(A)** Hippocampal Bax expression levels were significantly up-regulated in the MK-801-treated mice, while PF administration abolished this alteration. **(B)** PF administration significantly inhibited MK-801-induced Bax up-regulation in the prefrontal cortex. Olanzapine treatment in the model mice significantly down-regulated Bax levels in the hippocampus, but not in the prefrontal cortex. **(C)** 10 mg/kg and 50 mg/kg, but not 200 mg/kg PF treatment significantly increased tyrosine hydroxylase levels in the MK-801 model mice. Three-way ANOVA statistics: *F*_A_ = 48.86, *P* < 0.001; *F*_B_ = 26.87, *P* < 0.001; *F*_C_ = 11.15, *P* < 0.001. Values are the mean ± SEM, *n* = 3/group. *p < 0.05, **p < 0.01, ***p < 0.001.

We subsequently analyzed the apoptotic protein-Bax expression levels in the hippocampus and prefrontal cortex of mice exposed to different treatments. Western blot analyses of Bax levels in hippocampus and prefrontal cortex were shown in Figures 3A,B. Hippocampal Bax expression levels were significantly up-regulated in the MK-801-treated mice, while 10 mg/kg (*P* < 0.001), 50 mg/kg (*P* < 0.001) and 200 mg/kg (*P* < 0.001) PF administration abolished this alteration. Consistently, 10 mg/kg (*P* < 0.01), 50 mg/kg (*P* < 0.001) and 200 mg/kg (*P* < 0.01) PF administration significantly inhibited MK-801-induced Bax up-regulation in the prefrontal cortex. Interestingly, olanzapine treatment in the model mice significantly down-regulated Bax levels in the hippocampus, but not in the prefrontal cortex.

We also measured the expression of tyrosine hydroxylase in the prefrontal cortex of MK-801-treated mice. Western blot analyses showed that the MK-801-treated mice had significantly decreased tyrosine hydroxylase levels in the prefrontal cortex when compared with control mice, while 10 mg/kg (*P* < 0.05) and 50 mg/kg (*P* < 0.01), but not 200 mg/kg (*P* > 0.05) PF treatment significantly increased tyrosine hydroxylase levels in the model mice (Figure 3C).

### PF Protected Against Oxidative Stress-Induced Damage in Primary Cultured Hippocampal Neurons

**Figure 4:**
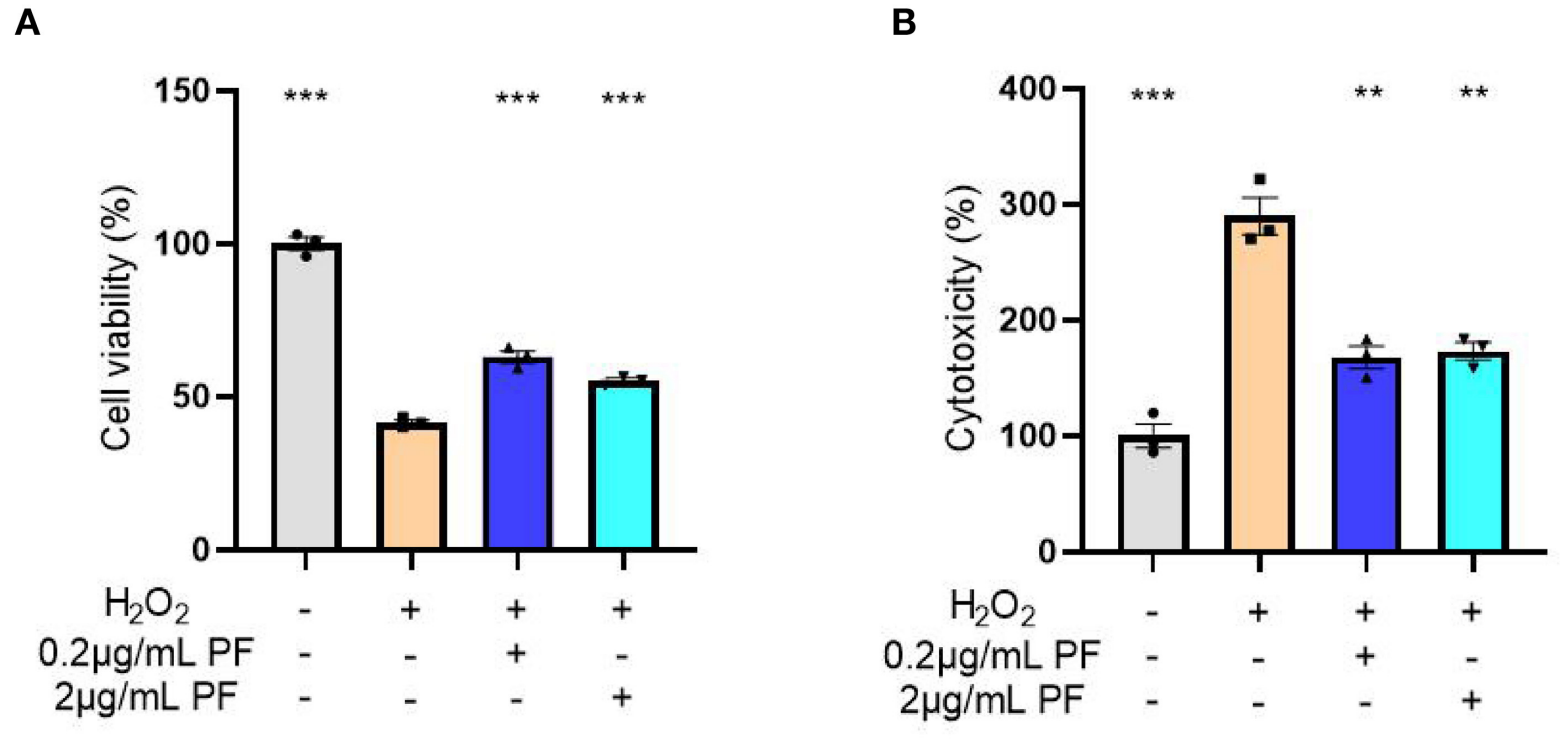
Paeoniflorin (PF) protected against oxidative stress-induced neurotoxicity in primary cultured hippocampal neurons. **(A)** The cell viability of primary cultured hippocampal neurons was markedly decreased after hydrogen peroxide treatment when compared with control group (*P* < 0.001). However, the severity of this decrease in cell viability was significantly reduced when the neurons were pretreated with 0.2 μg/ml (*P* < 0.001) and 2 μg/ml (*P* < 0.001) PF. **(B)** Analysis of LDH release, as a measure of cytotoxicity, demonstrated that hydrogen peroxide caused significantly increased cytotoxicity in the primary cultured hippocampal neurons, whereas treatment of 0.2 μg/ml (*P* < 0.001) and 2 μg/ml (*P* < 0.001) PF in the neurons significantly reduced the severity of the cytotoxicity. Three-way ANOVA statistics: *F*_A_ = 250.7, *P* < 0.001; *F*_B_ = 47.93, *P* < 0.001. Values are the mean ± SEM, *n* = 3/group. **p < 0.01, ***p < 0.001.

To directly evaluate the effects of PF on oxidative stress-induced damage, we used hydrogen peroxide to cause oxidative stress in primary cultured hippocampal neurons. As shown in Figure 4A, the cell viability of primary cultured hippocampal neurons was markedly decreased after hydrogen peroxide treatment when compared with control group (*P* < 0.001). However, the severity of this decrease in cell viability was significantly reduced when the neurons were pretreated with 0.2 μg/ml (*P* < 0.001) and 2 μg/ml (*P* < 0.001) PF. Consistently, analysis of LDH release, as a measure of cytotoxicity, demonstrated that hydrogen peroxide caused significantly increased cytotoxicity in the primary cultured hippocampal neurons, whereas treatment of 0.2 μg/ml (*P* < 0.001) and 2 μg/ml (*P* < 0.001) PF in the neurons significantly reduced the severity of the cytotoxicity, as shown in Figure 4B.

**Figure 5:**
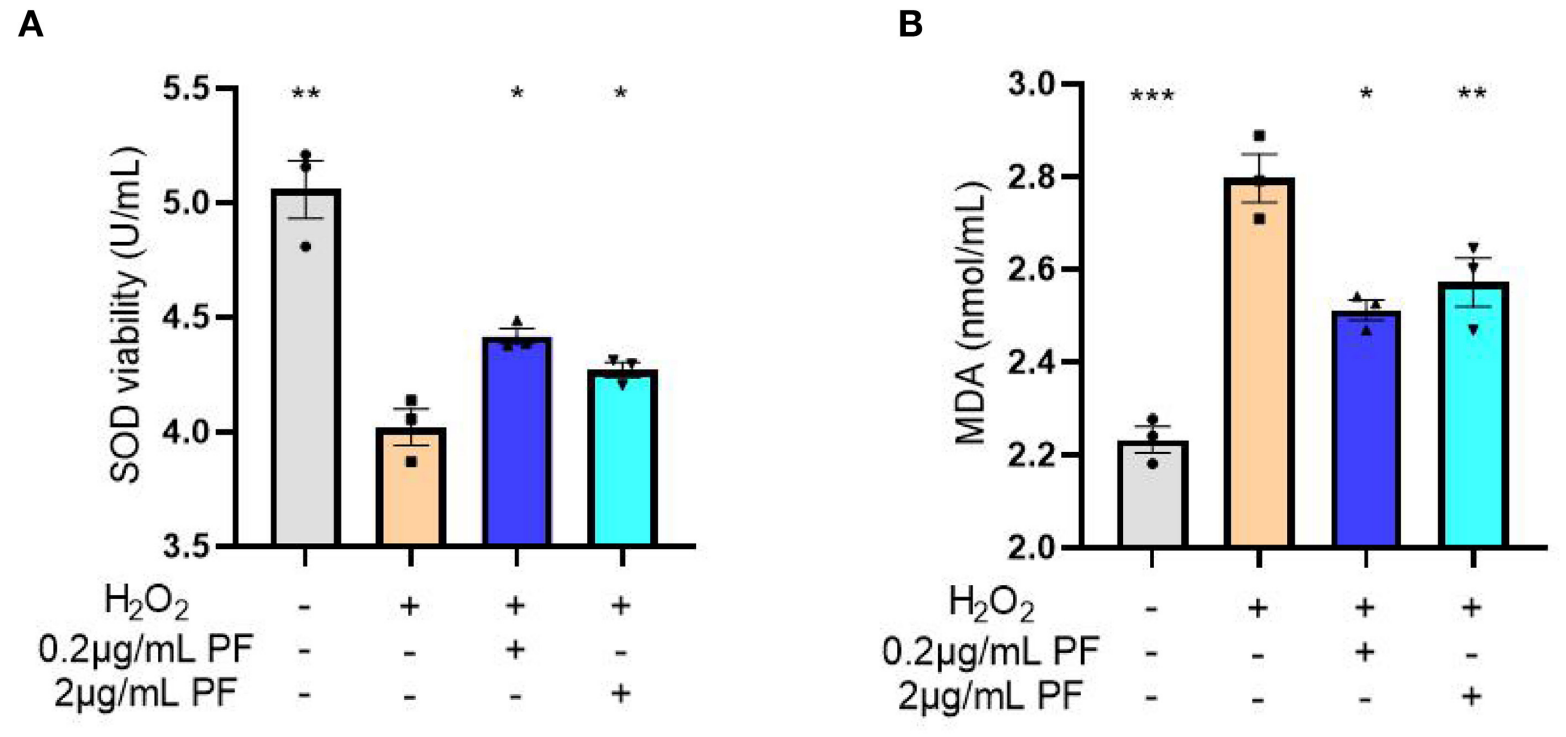
Paeoniflorin (PF) restored oxidative stress marker levels in the neurons exposed to hydrogen peroxide. **(A)** The superoxide dismutase (SOD) activities were significantly reduced after hydrogen peroxide exposure in the neurons (*P* < 0.01). However, pretreated with 0.2 μg/ml (*P* < 0.05) and 2 μg/ml (*P* < 0.05) PF in the damaged neurons significantly restored SOD activities. **(B)** The increased MDA levels in the primary cultured neurons caused by hydrogen peroxide exposure were significantly inhibited by pretreatment with 0.2 μg/ml (*P* < 0.05) and 2 μg/ml (*P* < 0.01) PF. Three-way ANOVA statistics: *F*_A_ = 32.13, *P* < 0.001; *F*_B_ = 31.76, *P* < 0.001. Values are the mean ± SEM, *n* = 3/group. *p < 0.05, **p < 0.01, ***p < 0.001.

Next, the SOD activities and MDA levels were measured in the condition medium of primary cultured hippocampal neurons exposed to various treatments. As expected, the SOD activities were significantly reduced after hydrogen peroxide exposure in the neurons (*P* < 0.01). However, pretreated with 0.2 μg/ml (*P* < 0.05) and 2 μg/ml (*P* < 0.05) PF in the damaged neurons significantly restored SOD activities, as shown in Figure 5A. Moreover, the increased MDA levels in the primary cultured neurons caused by hydrogen peroxide exposure were significantly inhibited by pretreatment with 0.2 μg/ml (*P* < 0.05) and 2 μg/ml (*P* < 0.01) PF (Figure 5B).

## Discussion

Our study demonstrated that PF ameliorated MK-801-induced schizophrenia–like behaviors in mice, these included sensorimotor gating deficits evaluated by the PPI test, memory deficits by the fear conditioning test and hyperactivities by the open field test. Further analysis suggested that the increased oxidative stress and apoptosis in the MK-801-treated mice were reversed by PF administration. The *in vitro* data provided direct evidence that PF protected against oxidative stress in neurons. Therefore, our study was the first to provide evidence of a potential antipsychotic role of PF in SCZ, and the effect was likely through oxidative stress pathway. The potential antipsychotic role of PF in SCZ is consistent with previous reports showing that natural products ameliorated MK-801-induced SCZ like behaviors in mice, these natural products included maslinic acid (24) and Kami-ondam-tang (25).

The crucial role of oxidative stress in the neuroprotective effect of PF in the MK-801 model of SCZ is plausible given the dysregulated oxidative stress markers found in patients with SCZ (15). Studies have suggested showed higher MDA levels (26) and lower SOD activities (27) in peripheral blood of SCZ patients, both of which supported the hypothesis that SOD and MDA in SCZ patients led to the imbalance between the production and clearance of reactive oxygen species. These were consistent with our results showing that in the blood of SCZ model mice, the SOD activities were significantly decreased and the MDA contents were significantly increased, while PF treatment restored the dysregulated oxidative stress markers in the MK-801-treated mice. Additionally, oxidative stress pathway has been linked to targets for SCZ prognosis and treatment. Data from Wei et al. (28) suggested that oxidative stress mediated particulate matter in the environment was associated with the recurrence of SCZ. On the other hand, several studies reported that natural antioxidant products such as omega-3, vitamin E and vitamin C, intervening and regulating the function of oxidation-system (29, 30), which alleviated the symptoms of SCZ.

The beneficial effect of PF in SCZ via oxidative stress pathway is further supported by the excellent anti-inflammatory and immune regulation abilities of PF (7). It has been shown that both oxidative stress and immune function changes were closely related to SCZ (31). When the oxidative stress system was disordered, abnormal reactive oxygen species caused inflammation and participated in the pathological process of SCZ (32). Furthermore, it is well known that excessive oxidative stress and inflammation lead to apoptosis. Therefore, it is reasonable to find increased expression of pro-apoptotic protein marker-Bax, and decreased expression of anti-apoptotic protein-tyrosine hydroxylase in the brains of MK-801-treated mice. The brain regions we tested included prefrontal cortex and hippocampus, which are believed to be important for the onset and/or development of SCZ (33). In fact, clinical data indicated that the loss of neurons in brains of SCZ patients, especially in the prefrontal cortex, was the basis of many core symptoms of SCZ and was closely related to the prognosis of the disease (34). Here we found that PF treatment may ameliorate MK-801-induced apoptosis *in vivo* and protected against oxidative stress-induced damage *in vitro*, further validating the antipsychotic role of PF in SCZ.

One advantage of this study is that we have compared the effects of PF with the first-line antipsychotics-olanzapine on the MK-801 model of SCZ. Our results showed that both PF and olanzapine rescued prepulse inhibition deficits and hyperactivities in MK-801-mice, suggesting that PF may have similar effects with olanzapine on treating the positive symptoms of SCZ patients. Interestingly, PF treatment rescued memory deficits in the SCZ model mice, whereas olanzapine did not affect memory functions in the model mice. These results are consistent with the fact that antipsychotics predominantly target positive symptoms and do not address other facets of SCZ (21), and given that cognitive impairments in SCZ patients are very difficult to treat, our findings therefore provides a potential effective target for treatment of cognitive impairments in SCZ. The potential of PF as an alternative drug for treatment on SCZ is further supported by our findings that PF restored apoptotic-related protein expressions in prefrontal cortex of SCZ model mice, whereas olanzapine did not have the effects, although both PF and olanzapine reduced oxidative stress in serum of MK-801-treated mice. However, it should be noted that our findings of a potential antipsychotic role of PF in SCZ is preliminary, which requires validations from future investigations, and hopefully with collaborations between researchers and clinicians.

In conclusion, our results were the first to provide evidence that PF rescued schizophrenia-like behaviors in mice, including positive symptoms and cognitive impairments. Furthermore, oxidative stress and apoptosis pathways were involved in the antipsychotic role of PF in the SCZ model mice, and therefore our study provides a novel strategy for treatment of SCZ.

## Data Availability Statement

The raw data supporting the conclusions of this article will be made available by the authors, without undue reservation.

## Ethics Statement

The animal study was reviewed and approved by The Animal Care and Use Committee of Minzu University of China.

## Author Contributions

YC conceived and designed this study. J-QL and XC performed the experiments. J-QL, XC, and YC analyzed and interpreted the data. J-QL drafted the manuscript with critical revisions from YC and XC. All authors contributed to the article and approved the submitted version.

## Funding

This study was supported by the National Natural Science Foundation of China (82071676 and 81703492), High-Level Hospital Development Program for Foshan Climbing Project, the Natural Science Foundation of Guangdong Province (2114050002827), and Foshan Science and Technology Innovation Project (2020001005608).

## Conflict of Interest

The authors declare that the research was conducted in the absence of any commercial or financial relationships that could be construed as a potential conflict of interest.

## Publisher's Note

All claims expressed in this article are solely those of the authors and do not necessarily represent those of their affiliated organizations, or those of the publisher, the editors and the reviewers. Any product that may be evaluated in this article, or claim that may be made by its manufacturer, is not guaranteed or endorsed by the publisher.
